# *Cryptosporidium* spp. infection drives distinct alterations in the faecal extracellular vesicles metaproteome of calves

**DOI:** 10.1186/s40104-025-01332-4

**Published:** 2026-01-15

**Authors:** Chanaka Premathilaka, Kasun Godakumara, Mandy Jayne Peffers, Emily J. Clarke, Elisabeth Dorbek-Sundström, Toomas Orro, Suranga Kodithuwakku, Alireza Fazeli

**Affiliations:** 1 Institute of Veterinary Medicine and Animal Sciences, Estonian University of Life Sciences, Tartu, Estonia; 2 Department of Musculoskeletal and Ageing Science, Institute of Life Course and Medical Sciences, University of Liverpool, Liverpool, UK; 3 Department of Animal Science, Faculty of Agriculture, University of Peradeniya, Peradeniya, Sri Lanka; 4 Department of Pathophysiology, Institute of Biomedicine and Translational Medicine, Faculty of Medicine, Tartu University, Tartu, Estonia; 5 Division of Clinical Medicine, School of Medicine & Population Health, University of Sheffield, Sheffield, UK

**Keywords:** Calves, Cryptosporidiosis, Faecal extracellular vesicles, Gut-microbiome, Proteomics

## Abstract

**Background:**

The gut is primarily responsible for digestion and nutrient absorption, plays essential roles in immune regulation and metabolic balance, and is supported by a diverse microbiome essential for digestion, absorption, and defence from pathogens. Understanding gut physiology and pathophysiology in pre-weaned calves is essential, as infections like cryptosporidiosis can lead to gut dysbiosis, impair growth, and negatively affect long-term productivity. Faeces are considered easily accessible biological specimens that can be used to monitor gastrointestinal disorders. The methods employed in this study aimed to investigate the potential use of faecal extracellular vesicles (fEVs) as a non-invasive tool for assessing gut health and infections in calves. Particularly, considering Cryptosporidiosis as a model for gut infectious disease.

**Results:**

The analysis using a hybrid reference-based metaproteomic approach revealed that the proteomic profiles of fEVs significantly differed from that of faecal crude (FC) suspensions. Both sample types contained microbial and host proteins, which are important for maintaining gut defence and microbial homeostasis. However, *Cryptosporidium* spp. infection significantly shifted the fEV proteome, reducing both host and microbial proteins involved in gut defence. It also reduced proteins from microbes that are important for maintaining microbial homeostasis, while increasing stress-related proteins. Further, lyophilisation of fEVs significantly altered the protein profiles.

**Conclusion:**

These findings underscore that fEVs contain host and microbial proteins that are a valuable resource for studying gut physiology, pathophysiology, host-microbe-pathogen interactions, and microbiome dynamics. Changes in the proteomic profile of fEVs during *Cryptosporidium* spp. infection demonstrates the pathogen’s ability to manipulate host immune defences and microbiome composition for its survival and replication. Overall, these findings support the utility of fEV proteomics as a non-invasive platform for biomarker discovery and advancing research in gastrointestinal health and disease in livestock.

**Supplementary Information:**

The online version contains supplementary material available at 10.1186/s40104-025-01332-4.

## Introduction

The gastrointestinal (GI) tract is not only responsible for digestion and nutrient absorption, but it also plays a pivotal role in maintaining overall health, acting as a mediator for immune system regulation, maintaining metabolic balance, and supporting a diverse and dynamic microbial ecosystem. Furthermore, GI microbes contribute to vital functions such as digestion, vitamin production, and defence against pathogens. Therefore, gut health is directly linked with the development and production of farm animals [1]. Gastrointestinal dysbiosis in young calves can reduce the growth and future productivity of farm animals. Especially the infectious diseases, such as cryptosporidiosis, can alter the gut microbiome and reduce the weight gain of calves [2].

Faeces are an easily accessible biological specimen that can be used to monitor GI disorders remotely and non-invasively [3]. In addition to waste products, faeces carry extracellular vesicles (EVs) potentially released by the host, gut microbiota, gut pathogens, and the diet of the animal. EVs play a crucial role in intercellular, interspecies, and interkingdom communication, which are especially important for maintaining gut homeostasis [4, 5]. Thus, the cargo carried by faecal derived EVs (fEVs) may provide a valuable platform for monitoring gut health and disease diagnosis [6]. Previous studies demonstrated that EVs present in the gut facilitate communication between the microbiota and host cells, influencing key processes such as immune modulation, epithelial barrier integrity, and pathogen defense [4]. A study by Park et al. [7] showed that the metagenomic profile of human fEVs was altered in colorectal cancer patients. Further studies illustrated that fEV-associated proteins could serve as effective biomarkers to diagnose colorectal cancer [8]. This highlights the potential of using fEV-associated proteins to study gut pathology and pathophysiological changes, particularly during gut disorders. ​EVs enriched from faeces provide valuable biological material for studying host–pathogen communication [9–13].

Cryptosporidiosis is a zoonotic infection caused by protozoan parasites of the *Cryptosporidium* spp., and represents a significant global health challenge. It primarily impacts the GI system, resulting in millions of reported cases annually [14] and affects both humans and animals. Faecal samples are commonly used as biological specimens for the clinical and laboratory investigation of *Cryptosporidium* spp. infections [15]. Traditional approaches, such as 16S rRNA sequencing or deep sequencing-based metagenomics, provide valuable insights into the taxonomic composition of the gut microbiota during infection. However, they do not reveal the functional state of the microbial community [16], and the lack of information on many microbial proteins in the current databases poses challenges. Thus, alternative approaches like metaproteomics, particularly with customized databases integrated with other omics approaches (Multi-Omics Integration, Metagenome-Informed Metaproteomics), enable direct or indirect characterisation of the expressed proteins. Even though these models are not perfect, they can still help in linking microbial composition to functional activities to a greater extend [17, 18], allowing the detection of functional dysbiosis. Such functional insights are especially relevant for gastrointestinal infections like cryptosporidiosis, where the parasite’s interaction with both the host and microbiome can disrupt metabolic pathways, immune modulation, and barrier function [16, 17]. By capturing these functional changes, fEV proteomic profiling potentially offers a more comprehensive understanding of the pathophysiology of *Cryptosporidium* spp. infection, providing context that cannot be obtained from faecal microbial taxonomic compositional analyses alone.

In a recently published study, we successfully optimised a method to enrich fEVs from calf faeces with high purity [6]. These vesicles were shown to carry a cargo of protein, DNA, and RNA, making them excellent candidate biomarkers. These findings highlight the potential of using fEVs enriched from calf faeces as a non-invasive tool for diagnostics, prognostics, and monitoring gut infectious diseases [6].

Preserving biological samples, specially EVs for diagnostics and transportation, remains challenging due to the loss of bioactivity. Conventional methods utilizing liquid nitrogen or dry ice pose safety, cost, and logistical issues. Lyophilisation (freeze-drying) may offer a practical alternative, enable room-temperature storage while preserving EV cargo integrity [19]. Therefore, assessing the effect of lyophilisation on fEV proteomes is important to evaluate its suitability as a preservation method for future veterinary and biomedical applications.

In the current investigation, we investigated the proteomic profile of faecal crude suspension (FC) and enriched fEVs from pre-weaned calves. We assessed the impact of GI infections on the changes in the proteomic profiles of fEVs and compared the proteomic profiles of fEVs enriched from healthy and *Cryptosporidium* spp. infected samples as a readout for potential diagnostic biomarker development. Furthermore, the effect of lyophilisation on the proteomic profiles of enriched fEVs was also investigated.

## Materials and methods

### Animals and ethical clearance

The experiments involving animals were approved by the Ethical Committee of Animal Experiments of the Estonian Ministry of Agriculture (Animal Project Permit No. 116). Estonian Holstein–Friesian *(Bos taurus)*, 10-day-old female calves (*n* = 7) included in this study were all from the same large dairy farm located in Tartu County in South-East-Estonia. Calves were separated from their mothers immediately after the birth and fed 3 L of unpasteurized colostrum collected from the dam in the first 2 h following their birth. The animals were fed 2–3 kg of warmed, unpasteurized raw milk twice a day with free access to hay and starter feed (Prestarter, Agrovarustus OÜ, Tartu, Estonia). Direct rectal faecal samples from the calves were taken at 10 days of age for the experiments conducted in this study.

### Sample collection and initial laboratory analysis

Faecal samples were collected directly from the rectum using disposable gloves and immediately transported on ice to the laboratory. As described in our previous study [6], *Cryptosporidium* spp. and *Giardia* spp. infections were diagnosed by employing a commercial immunofluorescence-based staining kit (Crypto/Giardia Cel, Cellabs Pty Ltd., Sydney, Australia) according to the manufacturer’s instructions. Cysts and oocysts within the samples were visualised at 200 × magnification and categorised as none (no cysts/oocysts found), low (1–5 cysts/oocysts), medium (6–30 cysts/oocysts), and high (> 31 cysts/oocysts). Rotavirus and bovine coronavirus (BCV) infections were also tested using an ELISA method (Duo Digestive Kit, Bio-X, Jemelle, Belgium) according to the manufacturer’s guidelines.

Clinical evaluations were conducted at the time of sampling to assess the health status of each calf. Calves classified as healthy were confirmed negative for *Cryptosporidium* oocysts by immunofluorescence staining, whereas the infected group demonstrated a median oocyst count of 2.86 × 10^6^ oocysts per gram (OPG), with counts ranging from 2.37 × 10^5^ to 9.02 × 10^6^ OPG. None of the animals exhibited obvious clinical symptoms of cryptosporidiosis, except for one infected calf that showed mild diarrhea. Based on infection status and proteomic analyses, faecal crude samples collected from healthy animals were designated as FC, faecal extracellular vesicles enriched from healthy animals as fEV-H, and those enriched from infected animals as fEV-I.

### Preparing faecal crude suspension (FC) and enrichment of faecal extracellular vesicles (fEVs) for proteome analysis

The enrichment of fEVs was conducted as described in previous studies [6, 13]. In brief, 0.5 g of faecal sample was mixed with 10 mL of PBS (Dulbecco’s phosphate-buffered saline without Ca^2+^ and Mg^2+^, PBS, Verviers, Belgium) by vigorous vortexing. Then the samples were preprocessed by subjecting them to sequential centrifugations at 4 °C. First, 300 × *g* for 10 min to remove the undissolved particles, then the pellets were discarded, and the supernatants were used for another round of centrifugation at 400 × *g* for 10 min to remove cells. The resulting supernatants were centrifuged again at 4,000 × *g* for 10 min to remove any remaining cell debris. Finally, to further remove the particles that lie within the range of the apoptotic bodies, supernatants from the previous step were centrifuged at 10,000 × *g* for 10 min. The collected faecal supernatant was filtered through a polyethersulfone 0.45 µm filter (Minisart syringe filter, Gottingen, Germany). The preprocessed FC was used for proteomic analysis. For fEV enrichment, preprocessed samples were concentrated to 500 μL with Amicon Ultra-15 centrifugal filter devices (10 kDa cutoff, MerckMillipore, Darmstadt, Germany) and EVs were enriched using double size exclusion chromatography (SEC) in a cross-linked 4% agarose matrix of 90 μm beads (Sepharose 4 Fast Flow, GE HealthCare Bio-Sciences AB, Uppsala, Sweden) using a 10-cm gravity column calibrated with PBS. The fEV-enriched fractions 5 to 9 collected from the second SEC step were pooled and concentrated to 500 µL using Amicon^®^ Ultra-2 mL centrifugal filters (10 kDa cutoff, MerckMillipore, Darmstadt, Germany).

### Characterisation of fEVs

Characterisation of the enriched fEVs was performed as described previously [6, 13] based on the guidelines from the International Society for Extracellular Vesicles [20]. In brief, both the size and concentration of nanoparticles in the EV fractions were measured using Nanoparticle Tracking Analysis (NTA) ZetaView^®^ (PMX 110 V3.0 instrument by Particle Metrix GmbH, Inning am Ammersee, Germany, software version 8.05.14 SP7) coupled with ZetaView NTA software for data analysis. Transmission electron microscopy (TEM) was used for the morphological characterisation of the EVs. The enrichment of EV was confirmed by performing Western blotting of EV-specific surface protein markers CD63, internal marker TSG101, and purity marker Calnexin as reported in the previous study [6]. Additionally, liquid chromatography/mass spectrometry-mass spectrometry (LC–MS/MS) results of FC samples compared to enriched EVs were analysed to identify EV-specific protein markers and associated proteins following the International Society for Extracellular Vesicles guidelines [20].

### Lyophilisation of samples

A volume of 150 µL of enriched fEV sample was aliquoted into a 1.5-mL safe-lock polypropylene microcentrifuge tube and frozen overnight at −80 °C. Immediately before loading into a 2.5 L, −50 °C Benchtop Freeze Dryer (Labconco Corporation, Kansas, MO, USA), the tubes were opened, and a film in which 6 holes were pierced (1 mm diameter each) was placed on top of the tube’s opening. The samples were loaded into the freeze dryer, with a condenser temperature down to −55 °C and a vacuum pump capable of reaching an absolute pressure of 0.039 mBar. Samples were lyophilized for 10 h (with the environmental temperature conditioned to 22 °C) and stored at room temperature (23–25 °C) in a tightly sealed box to prevent moisture absorption and light exposure.

### Protein extraction

Lyophilised samples were reconstituted in 150 μL of PBS. Both lyophilised and non-lyophilised fEV samples (150 μL) were then suspended in 200 μL of urea lysis buffer (6 mol/L Urea, Sigma-Aldrich, Dorset, UK), 1 mol/L ammonium bicarbonate (Fluka Chemicals Ltd., Gillingham, UK) and 0.5% sodium deoxycholate (Sigma-Aldrich, Dorset, UK)). Samples were sonicated at 5 μm for 3 × 10 s per sample, with 1 min rest on ice between each sonication round.

### Bead-based protein extraction and digestion

Volumes of 340 μL of lysed and sonicated fEVs (~ 10 µg) and 160 μL of FC supernatant (~ 100 µg digested in solution) were treated with 10 μL of 1% (w/v) RapiGest SF Surfactant (Waters, Manchester, UK) (freshly prepared in 25 mmol/L ammonium bicarbonate (AmBic; Fluka Chemicals Ltd., Gillingham, UK). DL-Dithiothreitol (Sigma-Aldrich) was added (3 mmol/L final concentration) and incubated at 60 °C for 10 min. Iodoa-cetamide (Sigma-Aldrich) was added (9 mmol/L final concentration) and incubated at room temperature for 30 min in the dark. To each sample, 12 μL hydrophilic and hydrophobic magnetic carboxylate SpeedBeads (SP3 beads, total of 12 μL; Cytiva, Massachusetts, USA) were added, followed by 120 μL ethanol (Sigma-Aldrich, Dorset, UK), and samples were incubated at 24 °C for 1 h. The beads were separated from the samples using a magnetic stand and washed three times with 180 μL 80% ethanol. They were resuspended in 100 µL of 25 mmol/L AMBIC (Fluka Chemicals Ltd., Gillingham, UK). The 2 µg trypsin/LysC (Promega) was added to each sample and incubated at 37 °C for 2 h on a rotating incubator, were pulse spun to collect all liquid, and an extra 2 µg of Trypsin/Lys-C was added to each sample. Samples were then incubated at 37 °C overnight in a rotating incubator, to achieve complete protein digestion. Beads were removed from the samples using the magnetic stand and the supernatants were acidified by adding 1 μL trifluoroacetic acid (Sigma- Aldrich, Dorset, UK).

### Tandem liquid chromatography–mass spectrometry (LC–MS/MS)

Data-dependent LC–MS/MS analyses were conducted on a QExactive HF Quadrupole-Orbitrap mass spectrometer (Thermo Scientific, Hemel Hempstead, UK) coupled to a Dionex Ultimate 3000 RSLC nano-liquid chromatograph (Thermo Scientific, Oxford, UK). Sample digests (500 ng of equal amount of protein) were loaded onto a trapping column (Acclaim PepMap 100 C18, 75 μm × 2 cm, 3 μm packing material, 100 Å) using a loading buffer of 0.1% (v/v) trifluoroacetic acid, and 2% (v/v) acetonitrile in water for 7 min at a flow rate of 12 μL/min. The trapping column was then set in-line with an analytical column (EASY-Spray PepMap RSLC C18, 75 μm × 50 cm, 2 μm packing material, 100 Å) and the peptides eluted using a linear gradient of 96.2% A (0.1% (v/v) formic acid):3.8% B (0.1% (v/v) formic acid in water:acetonitrile (80:20) (v/v)) to 50% A:50% B over 60 min at a flow rate of 300 nL/min, followed by washing at 1% A:99% B for 5 min and re-equilibration of the column to starting conditions. The column was maintained at 40 °C, and the effluent was introduced directly into the integrated nano-electrospray ionisation source operating in positive ion mode. The mass spectrometer was operated in data-dependent acquisition (DDA) mode, acquiring survey scans over an *m/z* range of 350–2000 at a mass resolution of 60,000 (FWHM at *m/z* 200). The maximum injection time was 100 ms, and the automatic gain control was set to 3 × 10^–6^. The 12 most intense precursor ions with charge states of between 2 + and 5 + were selected for MS/MS with an isolation window of 2 *m/z* units. The maximum injection time was 100 ms, and the automatic gain control was set to 1 × 10^–5^. Fragmentation of the peptides was by higher-energy collisional dissociation. Mass spectrometry data are available in ProteomeXchange Consortium via the PRIDE with the dataset identifier PXD068377.

### Construction of a customised proteome database for identifying protein groups

A genus-level faecal reference proteome data set was generated using 16S rRNA sequencing data and literature-based bovine gut taxonomic information [17, 21]. In brief, genus-level taxonomic profiles were first obtained from 16S rRNA gene amplicon sequencing data from 10-day-old calves (*n* = 62) that included all the samples used for the current study (Table S1). This list was then cross-referenced with and expanded using genera reported in prior calf microbiome studies [1, 22, 23]. This list was expanded to also include the host species (*Bos taurus*), common protozoan pathogens affecting calves (*Cryptosporidium* spp. and *Giardia* spp.), and relevant viral pathogens (rotavirus and bovine coronavirus) [24–26]. Next, for all identified genera, including microbes, host, protozoa, and viruses, all available protein sequences were retrieved in FASTA format from UniProtKB, incorporating both reviewed (Swiss-Prot) and unreviewed (TrEMBL) entries. This composite dataset was then used to construct a custom reference database. Protein groups were identified using the large-scale proteomic analyser MaxQuant (version 2.7.3.0, Max Planck Institute of Biochemistry, Martinsried, Germany) [27], and the protein groups were identified using label-free quantification while matching the features between runs. For MaxQuant analysis, carbamidomethylation of cysteine was set as fixed modification and oxidation of methionine and N-terminally acetylated as variable modification. Proteins were considered identified if at least 3 peptides matched, and transfer of identifications between runs based on accurate mass and retention time was enabled. A q-value cut-off of 0.01 (1% false discovery rate (FDR)) was used as the threshold for identification. The other parameters were set as default.

Taxonomic assignment of proteins to genera was then performed by matching each identified protein group to its corresponding genus in this custom reference database. This approach ensured that the proteomic analysis captured bacterial, host, protozoal, and viral proteins, enabling functional characterisation of the gut environment in both healthy and infected calves.

### Differential protein abundance and bioinformatics analysis

Differential protein abundance between groups was determined with R software version 4.3.3 using the “DEP” package [28]. Data were normalised using Variance Stabilizing Normalization (VSN), and the missing values were imputed under a low-intensity assumption using the “man” (shift = 1.8, scale = 0.3) method. *P*-values from the pairwise contrasts were adjusted for multiple testing using the Benjamini–Hochberg method to control the FDR values [29]. Proteins were considered significantly differentially abundant if the fold change in protein expression was log2 > 1 or log2 < −1 and FDR < 0.05 between the two groups. Genus-level identification of protein groups was obtained directly from the MaxQuant output tables, where taxonomic annotations were assigned based on the reference database used for protein identification. The output file was analysed using R software. To address the sample effect, data were normalised by the relative abundance scaling method. This yielded comparable relative abundances across samples, while low-abundance genera (total sum < 5) were excluded to reduce noise. The normalised genus-level abundance table was subsequently used for taxonomic visualisation. The number of identified proteins belonging to different kingdoms was illustrated as a percentage of the total number of proteins. Bacterial genera were further categorised as Gram-positive, Gram-negative, or Gram-variable by retrieving Gram-stain information from previous studies, organism metadata available in the UniProt database and the BacDive metadatabase (https://bacdive.dsmz.de/) [30, 31].

Gene Ontology (GO) annotation were performed separately for host and microbial proteins for biological processes, cellular component, and molecular functions using ID mapping option of UniProt. GO terms assigned to significantly enriched proteins were then summarised by frequency, providing a descriptive overview of functional categories. Analyses were conducted comparing FC versus fEVs as well as fEV-H versus fEV-I.

### Experimental set up

This experiment was designed to investigate the proteomics profile of calf FC and fEVs and investigated the alteration of the proteomic profile of fEVs during gut infection of *Cryptosporidium*. Furthermore, to investigate the effect of lyophilisation on fEVs, we conducted a comparative analysis of the proteomics profile of lyophilised and non-lyophilised fEVs. Faecal samples were collected from 10-day-old calves and categorised into two groups based on clinical evaluations and laboratory testing. The healthy group included samples from clinically healthy calves confirmed to be free of *Cryptosporidium* spp., *Giardia* spp., rotavirus, and Bovine Corona Viruses (BCV) infections. The infected group included samples from calves exhibiting *Cryptosporidium* infection, confirmed by laboratory tests to be positive for *Cryptosporidium* but not infected with *Giardia* spp., rotavirus, or BCV. Pre-processed faecal crude suspension (*n* = 3), and fEVs enriched from the faeces of healthy calves (*n* = 3), fEVs enriched from *Cryptosporidium* infected calves (*n* = 4), and lyophilized fEVs enriched from healthy calves (*n* = 3) were subjected to LC–MS/MS based label-free quantification proteomics analysis. All LC–MS/MS analyses were performed using equal total protein input (500 ng per sample), allowing normalization by protein amount rather than sample volume or particle count. First, the differences in proteomics profiles between FC and fEVs enriched from healthy calves were compared to investigate the differences in protein composition and investigate findings using functional enrichment analysis. Second, the impact of *Cryptosporidium* infection on the proteomic composition of fEVs was investigated by comparing fEVs from healthy calves (fEV-H) and those from infected calves (fEV-I), aiming to identify disease-specific patterns of fEV proteome.

Lastly, the effect of lyophilization and storing samples at −80 °C on the proteomic profile of fEVs was assessed by comparing lyophilized fEVs (fEV-L), (*n* = 3) and non-lyophilized/stored at −80 °C fEVs (fEV-NL), (*n* = 3) enriched from the same healthy calves.

## Results

### fEV characterization

**Fig. 1:**
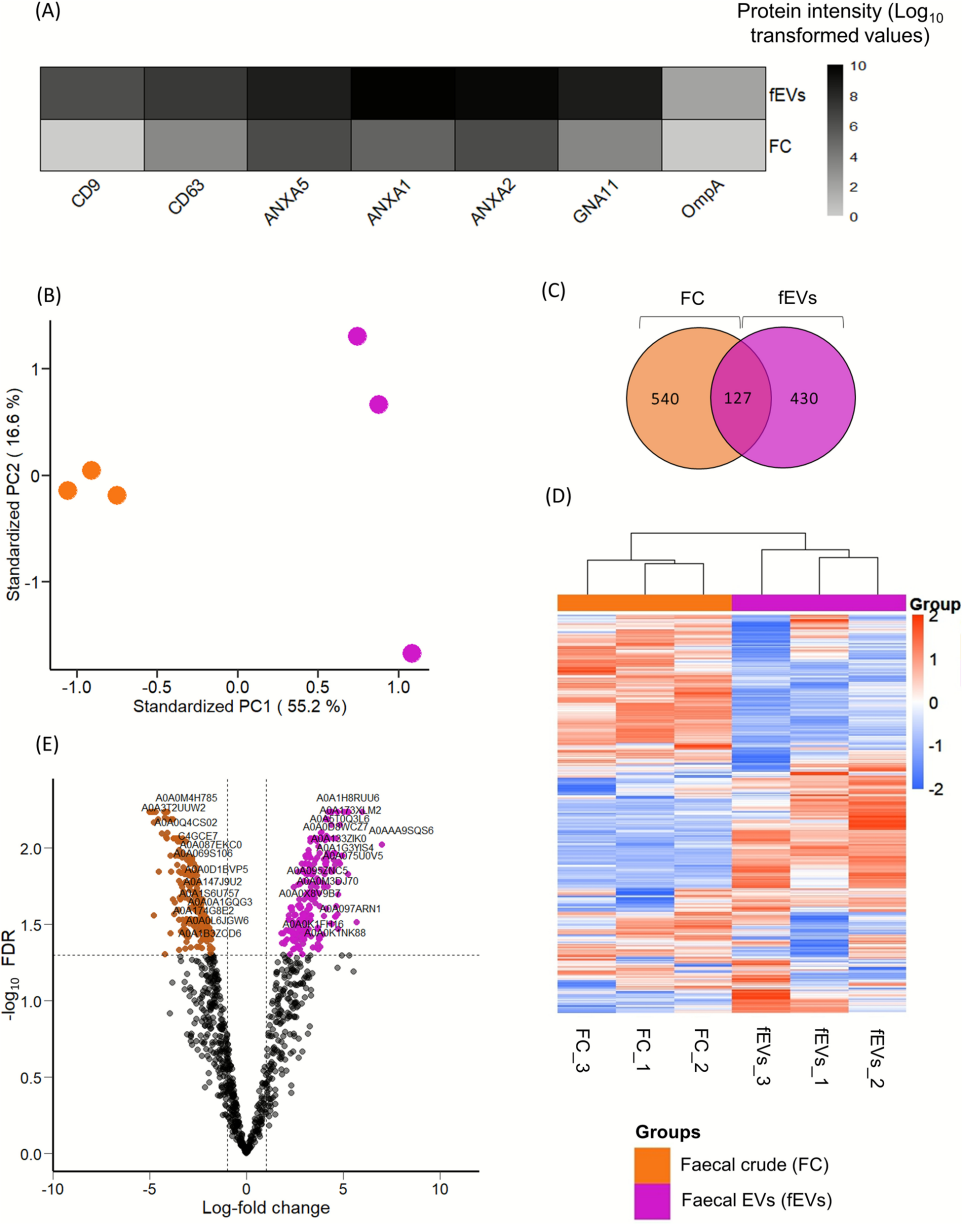
Protein profile of faecal crude suspension (FC) and faecal extracellular vesicles (fEVs) enriched from the same FC suspension sample collected from confirmed non-infected animals (*n* = 3). **A** Enrichment of EV-specific marker proteins such as tetraspanin (CD9, CD63), EV-associated proteins such as annexins (ANXA1, ANXA2, ANXA5), guanine nucleotide-binding protein subunit alpha-11 (GNA11), and outer membrane protein A (OmpA) in fEVs samples compared to FC. **B** Principal component analysis (PCA) illustrated the separation of samples based on differences in their overall proteomic profiles between two groups. **C** Venn diagram showing the number of distinct protein groups identified in FC and fEVs. **D** The heat map showed significant (*P* ≤ 0.05) differences in relative protein abundance between FC and fEVs. **E** Volcano plot comparing fEV versus FC showed that 236 proteins were significantly (*P* < 0.05) enriched and 201 proteins depleted in fEVs

The size distribution, morphology, concentration, and marker protein expression of the enriched fEVs used in this study were characterised in our previous work [6]. The average concentrations of fEVs in the fEV-enriched samples used for this study were 6.07 ± 2.16 × 10^11^ particles/mL in healthy samples and 4.39 ± 2.37 × 10^11^ particles/mL in infected samples. TEM analysis confirmed the vesicular morphology, and Western blotting detected EV markers CD63 and TSG101 while confirming the absence of the negative control marker calnexin. Additionally, the enrichment of EV specific protein markers such as tetraspanin (CD9, CD63), EV associated proteins/generic EV markers including annexins (ANXA1, ANXA2, ANXA5) and guanine nucleotide-binding protein subunit alpha-11 (GNA11), as well as Gram-negative bacterial EV associated protein outer membrane protein A (OmpA) were identified using LC–MS/MS analysis (Fig. 1A).

### Proteomic profiles of faecal crude suspension were different from fEV

Proteome comparison was conducted for both FC and fEVs (*n* = 3). Principal component analysis (PCA) revealed a clear distinction in the protein enrichment patterns between the faecal crude suspension and fEV protein profiles, with the first two principal components explaining 55.2%, and 16.6% of the total variance (Fig. 1B). Venn diagram analysis further showed 667 proteins in faecal crude suspension and 557 proteins in fEVs were identified, while 127 proteins were commonly detected for both groups. This demonstrates both the common protein core and the group-specific protein signatures distinguishing crude faeces from fEVs (Fig. 1C). Protein enrichment between FC and fEVs was indicated in the heat map (Fig. 1D). Furthermore, 236 proteins were significantly enriched (*P* ≤ 0.05) and 201 proteins were significantly reduced in fEVs compared to faecal crude samples (Fig. 1E).

### *Cryptosporidium* spp. infection altered the proteomic profile of fEVs

**Fig. 2:**
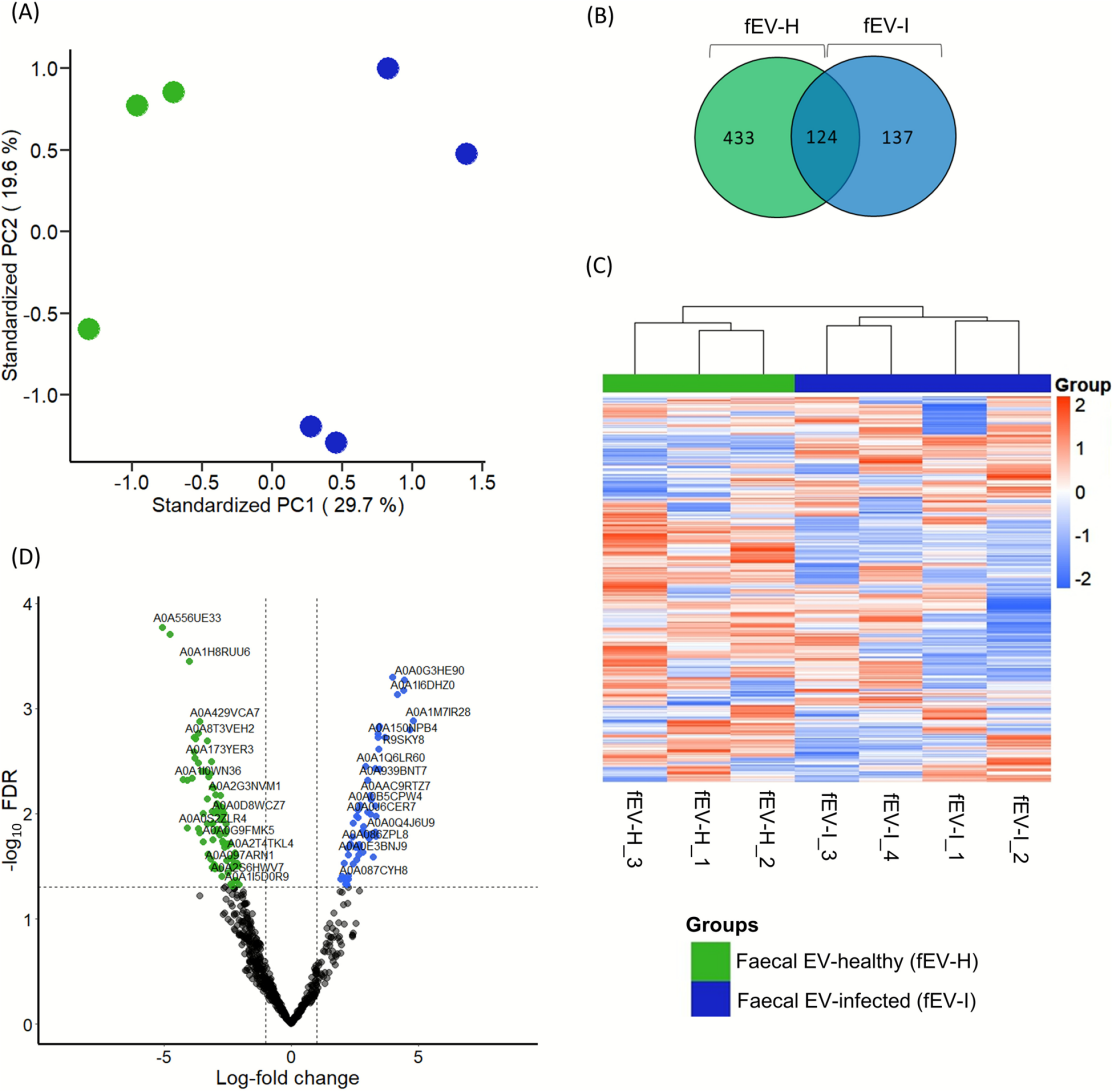
Protein profile of faecal extracellular vesicles enriched from the healthy group (fEV-H; *n* = 3) and *Cryptosporidium* spp. infected group (fEV-I; *n* = 4). **A** PCA analysis illustrated the separation of samples based on differences in their overall proteomic profiles between two groups. **B** Venn diagram of total proteins identified in fEVs enriched from healthy and infected groups, including those shared between groups. **C** The heat map showed significant (*P* ≤ 0.05) differences in relative protein abundance between fEV-H and fEV-I. **D** Volcano plot comparing fEV-I versus fEV-H showed that 54 proteins were significantly (*P* < 0.05) increased and 86 were reduced fEV-I compared to fEV-H

Proteomic profiling was performed in both fEV-H (*n* = 3) and fEV-I (*n* = 4) groups. PCA revealed a clear distinction in the protein enrichment patterns between the fEV-H and fEV-I protein profiles, with the first two principal components explaining 29.7% and 19.6% of the total variance (Fig. 2A). Using equal amounts of protein from each sample for LC–MS/MS analysis, the study identified 557 proteins in fEV-H and 261 in fEV-I groups. Out of these identified proteins, 124 proteins were common to both groups (Fig. 2B), and a proteomic profile of fEV-H is different from fEV-I (Fig. 2C). The differential enrichment analysis showed that 54 proteins were significantly enriched (*P* ≤ 0.05) and 86 proteins were significantly reduced in fEV-I compared to fEV-H (Fig. 2D).

### Lyophilization altered the fEV proteomic profile

**Fig. 3:**
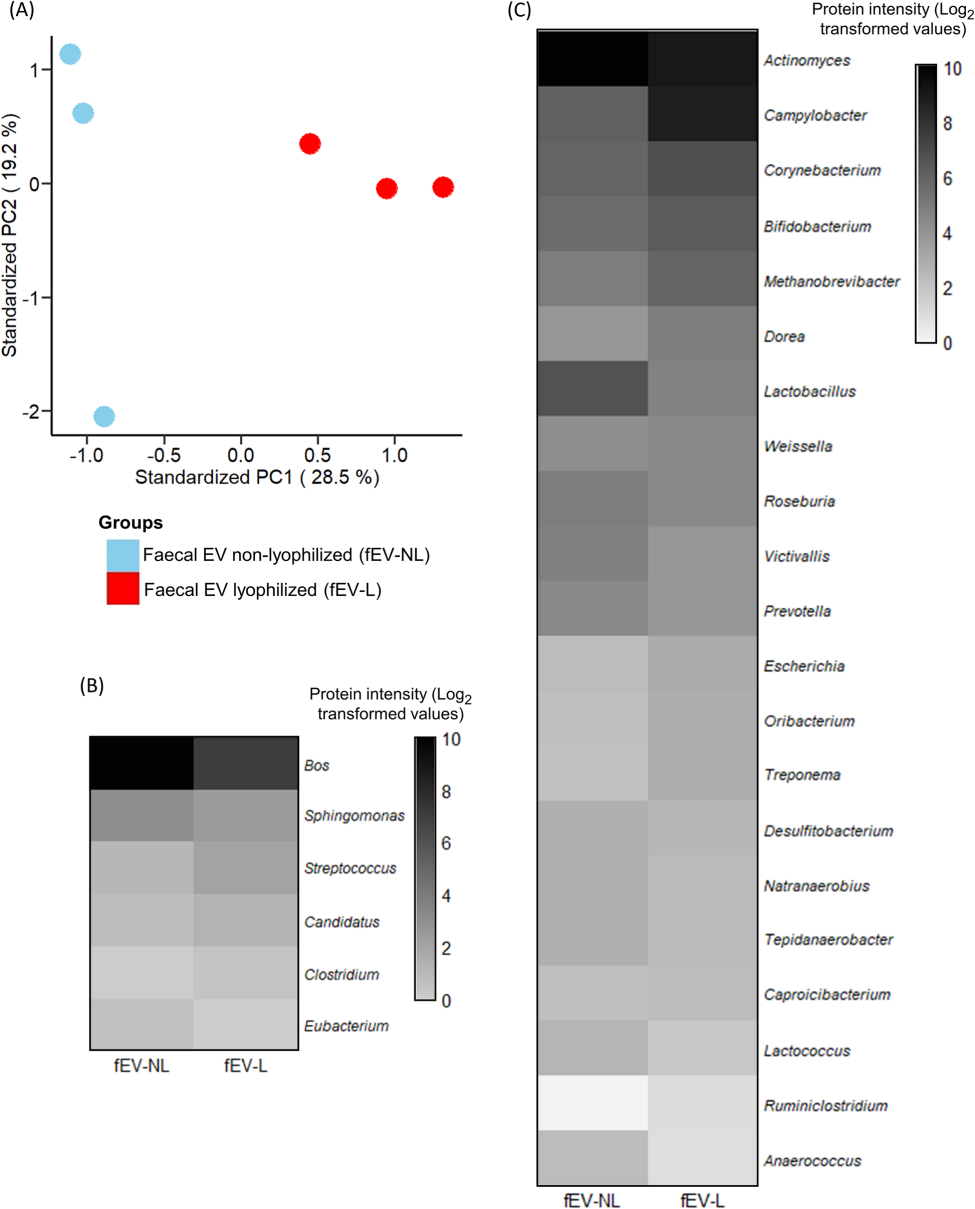
Protein profile of non-lyophilized faecal extracellular vesicles (fEV-NL) and lyophilized faecal extracellular vesicles (fEV-L) of the same samples (*n* = 3). **A** PCA illustrated the separation of samples based on differences in their overall proteomic profiles between two groups. **B** Relative protein intensities of the top six genera in fEV-NL and fEV-L. **C** Relative protein intensities of the next 20 genera in fEV-NL and fEV-L

Faecal EV samples containing average of 6.07 ± 2.16 × 10^11^ particles/mL concentration were used to study the effect of lyophilization on the proteomics profile of fEVs without any lyoprotectants (*n* = 3). PCA revealed a clear distinction in the protein enrichment patterns between the fEV-L and fEV-NL protein profiles, with the first two principal components explaining 28.5% and 19.2% of the total variance (Fig. 3A). This distinction was clearly indicated by the comparison of protein intensities between the two groups, which revealed differences in protein abundance across genera. Among the top six genera with the highest protein intensities, proteins from the host, *Sphingomonas*, and *Eubacterium* were more abundant in fEV-NL samples, whereas proteins from *Streptococcus*, *Candidatus*, and *Clostridium* were enriched in the fEV-L group (Fig. 3B). Extending the analysis to the next 20 genera also showed consistent differences in protein intensities between the two groups (Fig. 3C).

### Faecal crude suspension and fEVs contained proteins derived from different organisms

**Fig. 4:**
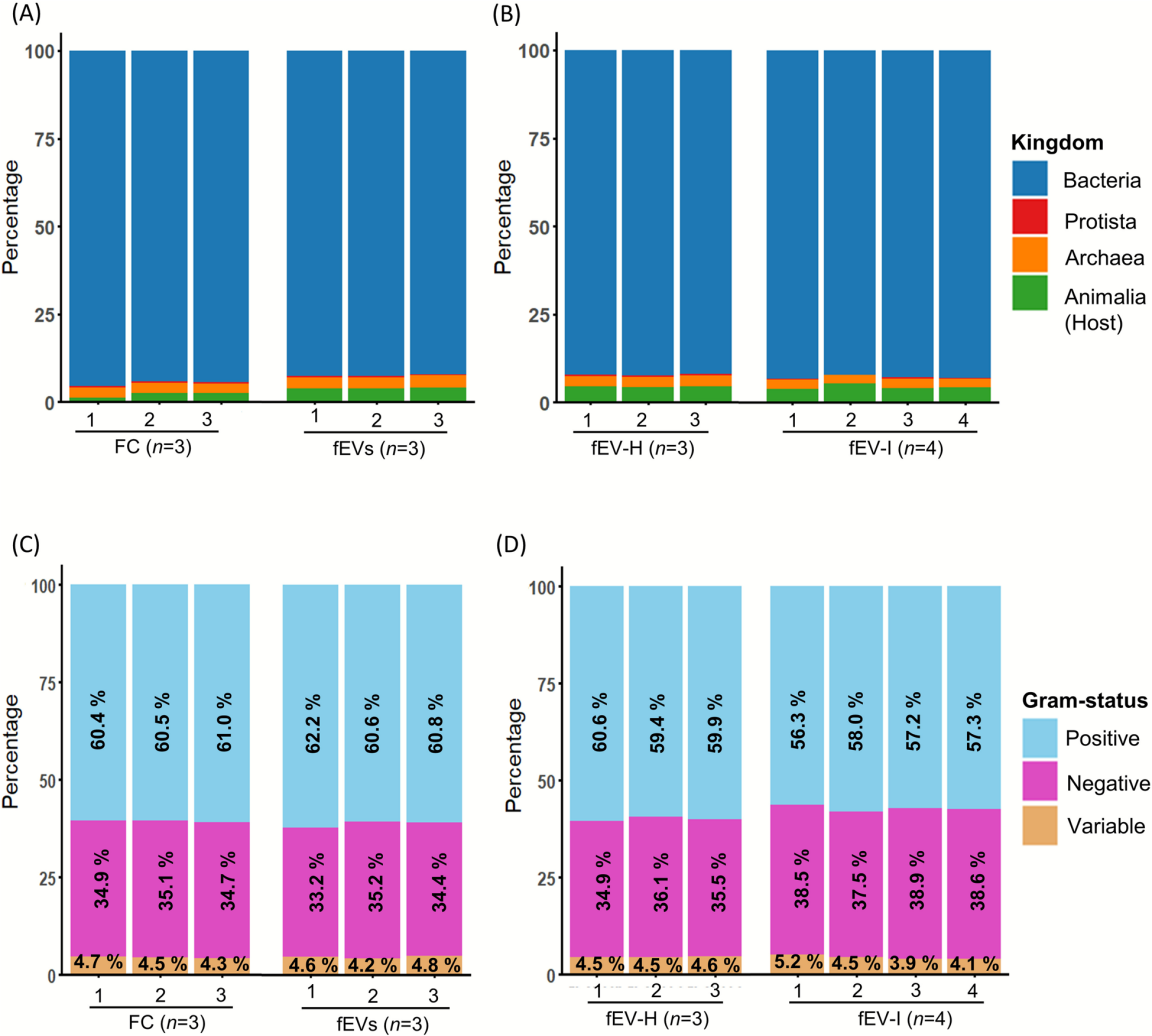
Origin of protein identified in FC and fEVs as a percentage of total protein count. Percentages of proteins originating from Kingdom Bacteria, Archaea, Protista, and host in individual samples as comparisons between (**A**) faecal crude suspension (FC) and faecal extracellular vesicles (fEVs), (**B**) faecal EVs-healthy (fEV-H) and faecal EVs-infected (fEV-I). The Gram staining characteristics of bacterial genera identified based on the proteomic profiles are displayed for individual samples of different studies (**C**) FC and fEVs comparison, (**D**) fEV-H and fEV-I comparison

Proteomic analysis of FC and fEV samples demonstrated the presence of proteins derived from the Bacteria, Archaea, Protista, and Animalia (host) kingdoms with variations in their relative proportions. The following percentages represent the number of identified proteins from each kingdom out of the total number of proteins detected in a given sample group. In the comparison between FC and fEVs from healthy samples, FC exhibited 94.7% ± 0.7% of proteins from Bacteria, 2.8% ± 0.1% from Archaea, 0.4% ± 0.08% from Protista, and 2.1% ± 0.8% from Animalia (host) as a percentage of total number of proteins based on the protein counts. Similarly, the fEVs group contained 92.5% ± 0.3% Bacteria, 3.2% ± 0.2% Archaea, 0.4% ± 0.07% Protista, and 4.0% ± 0.2% Animalia (host) proteins (Fig. 4A). Comparison between fEV-H and fEV-I groups demonstrated that the fEV-H group consisted of proteins from Bacteria 92.2% ± 0.2%, Archaea 3.1% ± 0.2%, Protista 0.3% ± 0.01%, and Animalia (host) 4.4% ± 0.1%. The fEV-I group displayed a distribution of 92.8% ± 0.5% from Bacteria, 2.6% ± 0.2% from Archaea, 0.3% ± 0.08% from Protista, and 4.4% ± 0.6% from Animalia (host) (Fig. 4B). All the groups contained proteins derived from both Gram-positive and Gram-negative bacterial genera. The genera include both Gram-positive and Gram-negative bacteria were categorized under Gram-variable genera. Both FC and fEV samples contained proteins from Gram-positive and Gram-negative bacteria, with no significant differences in their relative proportions between groups. (Fig. 4C). Similarly, comparison between healthy and infected calves showed no significant differences in the distribution of Gram classification of proteins (Fig. 4D).

### Proteomic profiles of FC and fEVs indicated changes in the relative abundance of proteins belonging to different genera

**Fig. 5:**
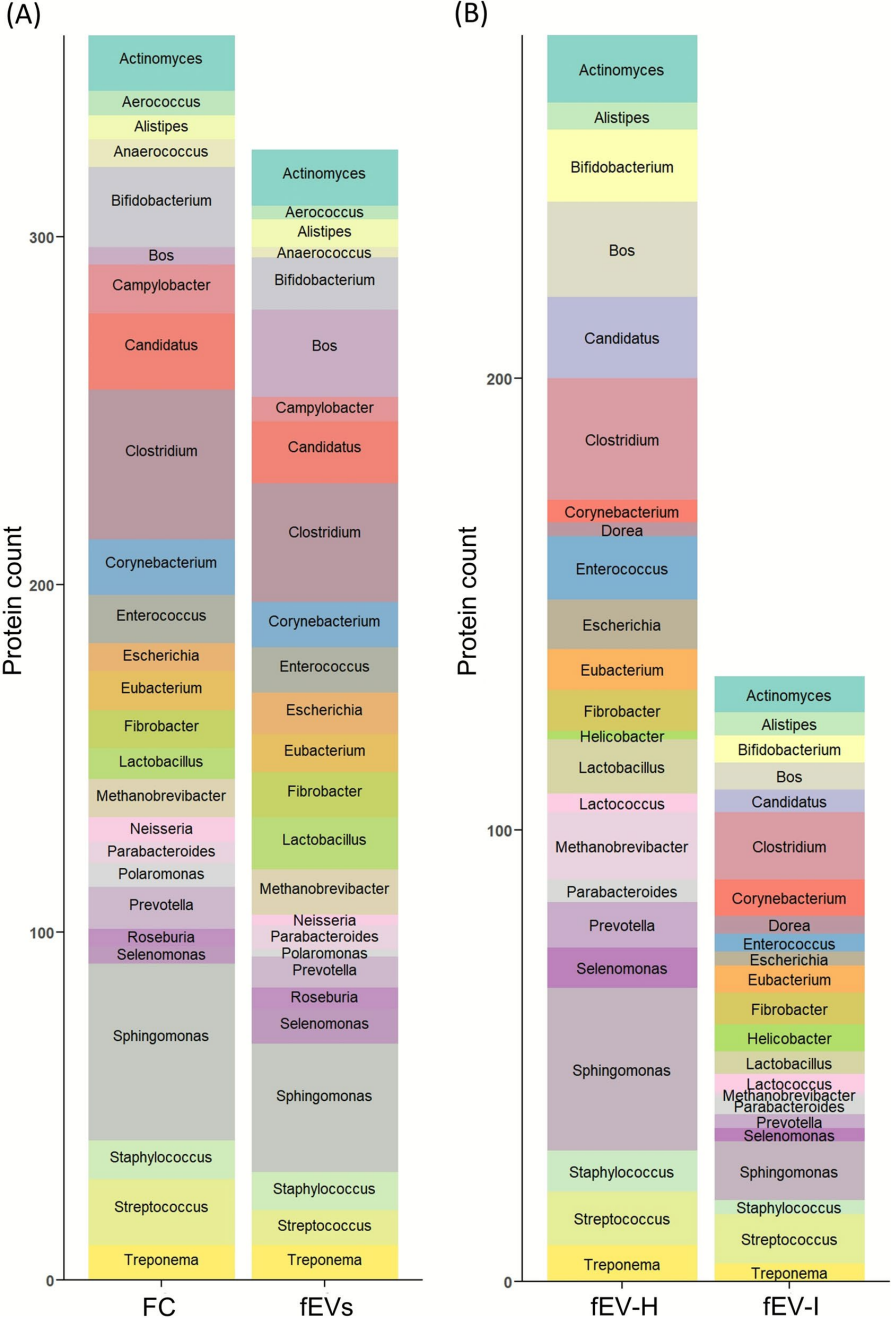
Illustration of top 10% genera-based protein counts. Comparison between (**A**) faecal crude (FC; *n* = 3), and faecal extracellular vesicles (fEVs; *n* = 3), (**B**) faecal EVs from healthy (fEV-H; *n* = 3), and *Cryptosporidium* spp. infected (fEV-I; *n* = 4) groups

Protein count were analysed across genera for each comparison, and the top 10% of genera were visualised in Fig. 5. In the comparison between FC and fEVs, 155 genera contributed to the protein pool of the FC group, while 144 genera contributed to the fEV group, with marked differences in their relative proportions. When considering top 10% abundance proteins, host-derived proteins from *Bos taurus*, microbial proteins from genera including *Lactobacillus*,* Fibrobacter*,* Methanobrevibacter*,* Escherichia*,* Selenomonas*,* Alistipes*, and *Parabacteroides* originated proteins were enriched in fEVs compared to FC (Fig. 5A). Interestingly, the host proteins, such as dipeptidase 1 (DPEP1), cadherin-related family member 5 (CDHR5), olfactomedin 4 (OLFM4), and polymeric immunoglobulin receptor (pIgR), all of which are associated with immune defense and maintenance of intestinal homeostasis. Additionally, proteins involved in mucosal secretion and barrier function, such as Fc gamma binding protein (FCGBP), Mucin 13 (MUC13), and Calcium-activated chloride channel regulator 1 (CLCA1), were significantly enriched in fEVs. For the fEV-H and fEV-I groups, 138 genera were identified in the fEV-H group compared to 104 genera in the fEV-I group. Except *Corynebacterium* and *Helicobacter*, all other genera show a decrease in the number of proteins in the fEV-I proteome compared to fEV-H (Fig. 5B). Interestingly, *Cryptosporidium* spp. derived proteins were not detected even in the fEV-I samples. Host derived proteins, including DPEP1, CDHR5, OLFM4, FCGBP, MUC13, and CLCA1 proteins were significantly reduced in the fEV-I group compared to fEV-H group.

### Gene Ontology (GO) annotation

To obtain GO functional annotation overview of the proteins, the significantly enriched proteins in each group, FC, fEVs, fEV-H, and fEV-I, were assessed using the descriptions extracted from each protein’s Uniprot entry. Gene Ontology categories for molecular function (MF), biological processes (BP), and cellular components (CC) were assessed by mapping 3 host proteins and 197 microbial proteins in FC, 10 host proteins and 225 microbial proteins in fEVs, 2 host proteins and 83 microbial proteins in fEV-H, and 54 microbial proteins in fEV-I. Notably, host proteins were not significantly enriched in the fEV-I group compared to fEV-H.

#### Gene Ontology annotation by molecular function (MF)

**Fig. 6:**
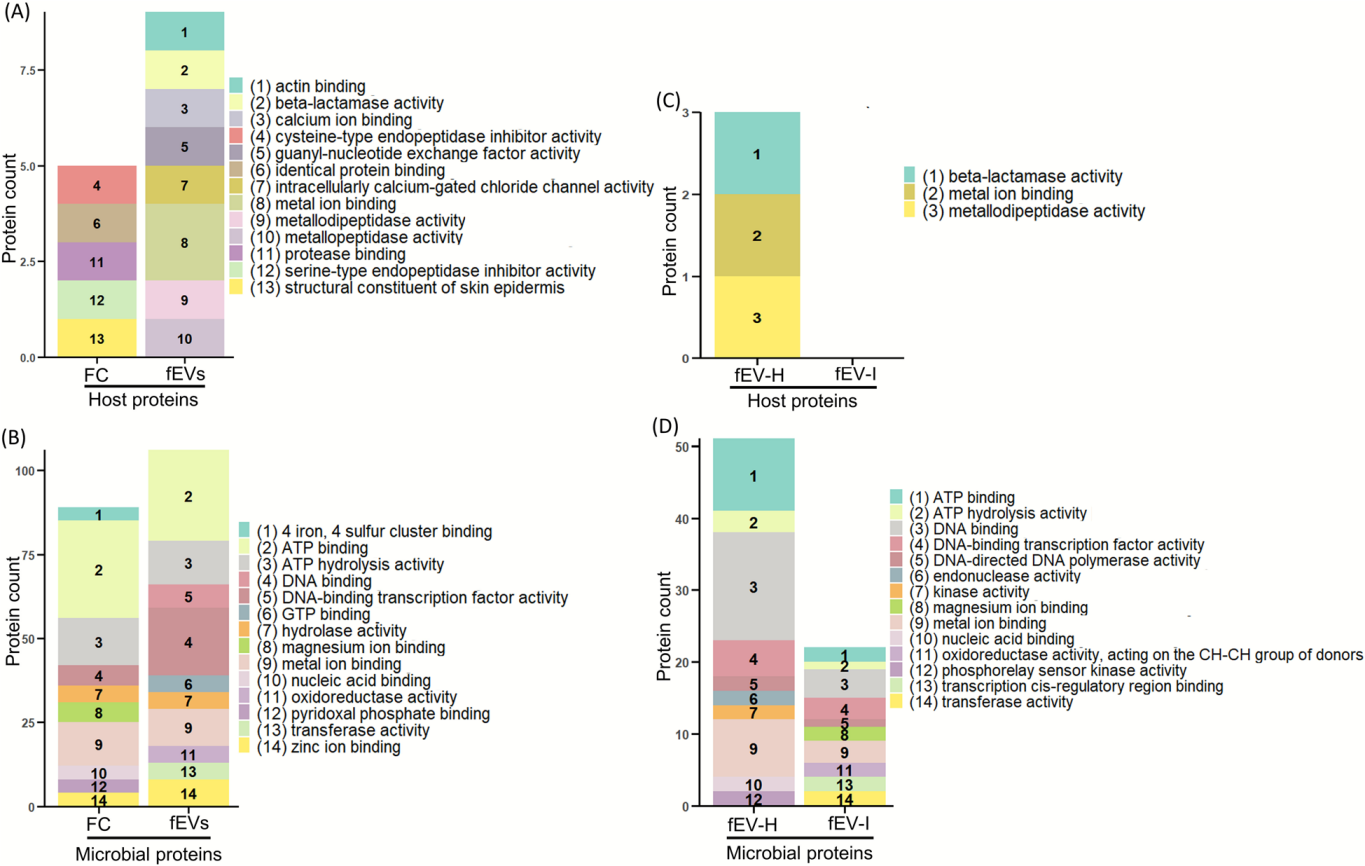
Gene Ontology (GO) annotation for molecular function. GO annotation of (**A**) host proteins present in faecal crude (FC; *n* = 3), and faecal EV (fEVs; *n* = 3), (**B**) microbial proteins of FC and fEVs, (**C**) host proteins of healthy group faecal EVs (fEV-H; *n* = 3), and *Cryptosporidium* spp. infected group faecal EVs (fEV-I; *n* = 4), (**D**) microbial proteins of fEV-H and fEV-I. Figure indicates the top 10 molecular functions of each group

The top 10 MFs revealed that the MFs identified for host proteins present in FC and fEVs were distinct from each other (Fig. 6A). For microbial proteins, functions related to energy metabolism, particularly ATP binding and ATP hydrolysis, were prominent MFs in both groups. Additionally, proteins related to MFs, such as DNA binding, zinc ion binding, DNA-binding transcription factor activity, GTP binding, oxidoreductase activity, and transferase activity were enriched in fEVs (Fig. 6B). Significant enrichment of host proteins was not observed in fEV-I compared to fEV-H, and consequently, MFs were not identified for fEV-I group (Fig. 6C). Microbial proteins annotated in magnesium ion binding, oxidoreductase activity, acting on the CH-CH group of donors, transcription *cis*-regulatory region binding, and transferase activity were more presented in fEV-I group. In addition, proteins associated with all other MFs also showed a decrease in fEV-I compared to fEV-H (Fig. 6D).

#### Gene Ontology annotation for biological processes (BP)

**Fig. 7:**
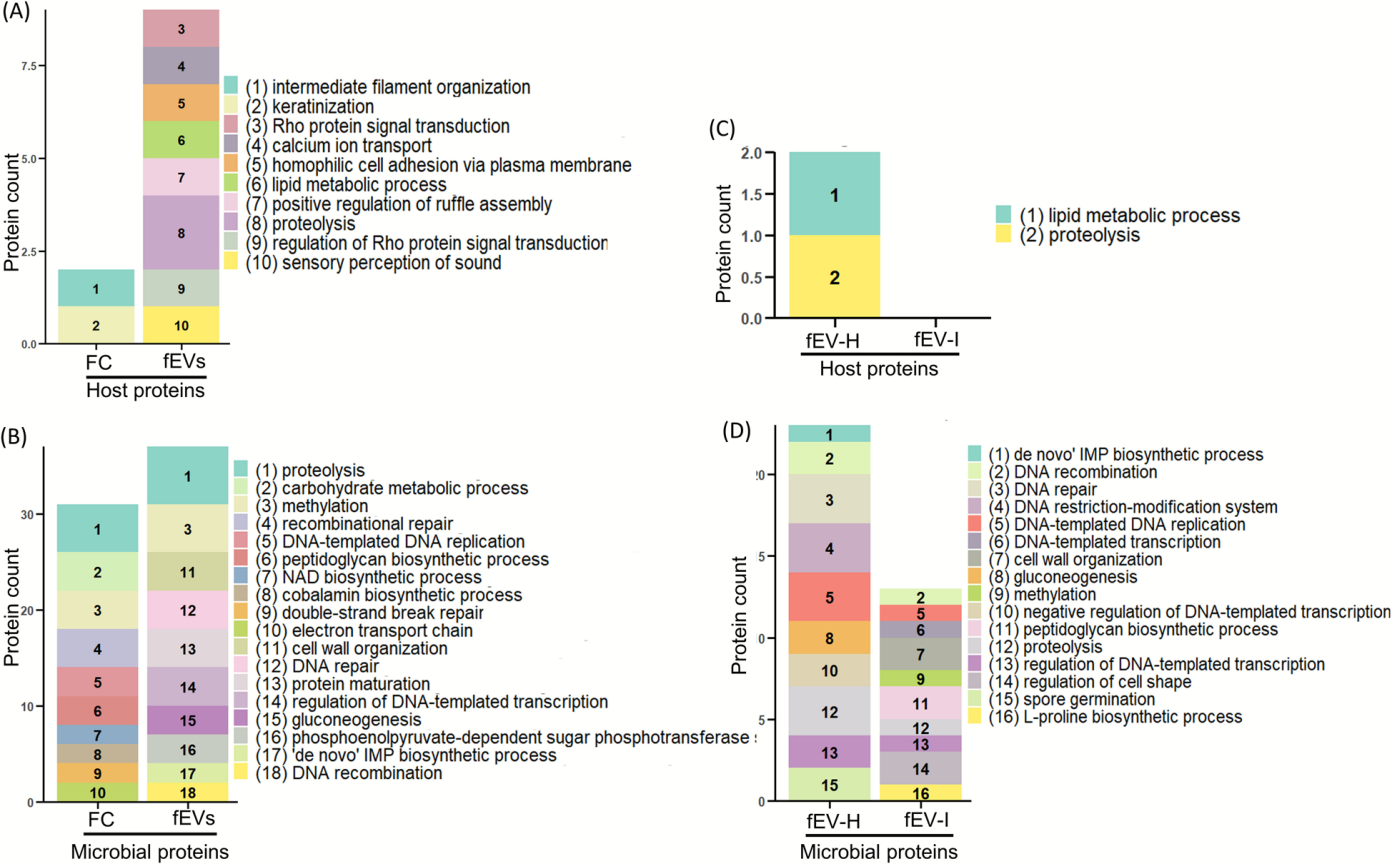
Gene Ontology annotation for biological processes. GO annotation of (**A**) host proteins present in faecal crude (FC; *n* = 3), and faecal EV (fEVs; *n* = 3), (**B**) microbial proteins of FC and fEVs, (**C**) host proteins of healthy group faecal EVs (fEV-H; *n* = 3), and *Cryptosporidium* infected group faecal EVs (fEV-I; *n* = 4), (**D**) microbial proteins of fEV-H and fEV-I. Figure indicates the top 10 molecular functions of each group

When considering the top 10% BPs of host proteins in FC and fEVs were distinct, with fEV proteins participating in a broader range of processes than those in FC (Fig. 7A). For microbial proteins, processes such as cell wall organization, DNA repair, protein maturation, regulation of DNA-templated transcription, gluconeogenesis, phosphoenolpyruvate-dependent sugar phosphotransferase system, ‘de novo’ IMP biosynthetic process, and DNA recombination were uniquely represented in fEVs (Fig. 7B). In the fEV-H and fEV-I comparison, host proteins in fEV-H were primarily annotated with lipid metabolic process, and proteolysis (Fig. 7C). Microbial proteins in fEV-I were annotated in processes including DNA-templated transcription, cell wall organization, methylation, peptidoglycan biosynthetic process, regulation of cell shape and L-proline biosynthetic process (Fig. 7D).

#### Gene Ontology annotation for cellular component (CC)

**Fig. 8:**
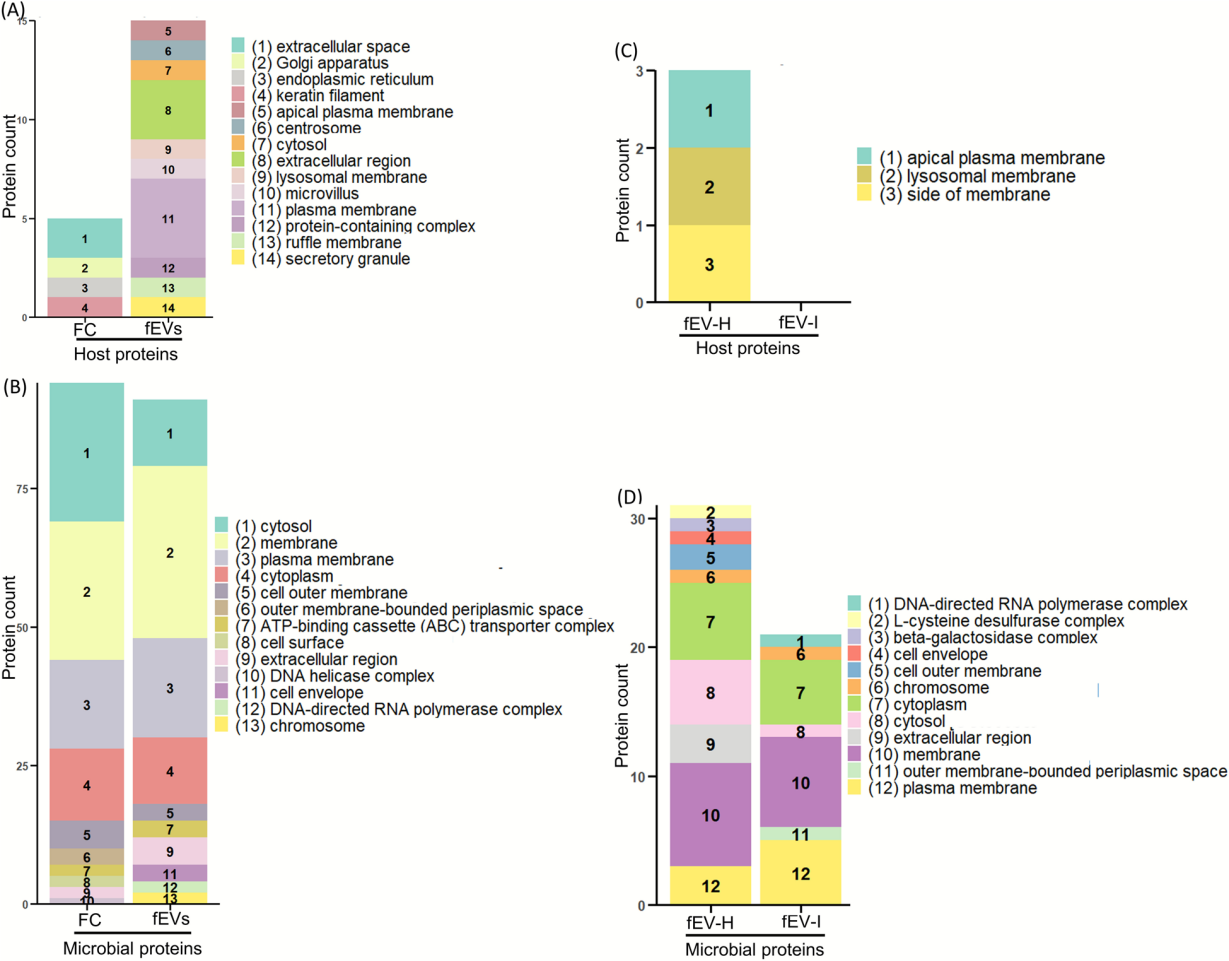
Gene Ontology annotation for Cellular Component. GO annotation of (**A**) host proteins present in faecal crude (FC; *n* = 3), and faecal EV (fEVs; *n* = 3), (**B**) microbial proteins of FC and fEVs, (**C**) host proteins of healthy group faecal EVs (fEV-H; *n* = 3), and *Cryptosporidium* spp. infected group faecal EVs (fEV-I; *n* = 4), (**D**) microbial proteins of fEV-H and fEV-I. Figure indicates the top 10 biological processes of each group

Comparison of top 10% CCs showed that host proteins in FC and fEVs were annotated with distinct CCs in each group (Fig. 8A). In the fEV group, plasma membrane proteins were predominantly represented. For microbial proteins, both FC and fEVs were enriched in membrane and plasma membrane proteins, although these were more abundant in fEVs (Fig. 8B). In contrast, cytosolic proteins were highly enriched in FC compared to fEVs. Further comparison between the fEV-H and fEV-I groups revealed that *Cryptosporidium* spp. infection reduced the CC related proteins in both host proteins (Fig. 8C) and microbial proteins (Fig. 8D). The findings reveal variability in cellular component representation among the sample groups.

## Discussion

Faecal material can be considered a highly complex and informative non-invasive biological specimen to study gut pathology and pathophysiology. Previous works demonstrated that faeces contain EVs released by host, gut parasites, and gut microbiome. The double membrane of EVs protect their cargo, including proteins, from proteases, nucleases, and harsh environmental conditions. Additionally, the charged corona of EVs is also involved in cargo delivery. This enables the recovery of intact proteins secreted by the host and GI tract organisms, preserving their natural structure and function. While fEVs are suggested to mediate inter-species communication in the GI tract, the role of their protein cargo in GI infections remains unclear [6, 32].

A major challenge in analysing faeces and fEV proteome is the accurate identification and taxonomic assignment of proteins within highly complex and diverse microbial communities. Although microbial proteomes are well represented in public databases such as UniProt, many entries remain unreviewed or poorly annotated, and the vast size and redundancy of these databases can reduce search efficiency and specificity. To overcome these challenges, we employed a customised protein database that has been optimised for our specific samples. This database combined protein sequences from both the reviewed (Swiss-Prot) and unreviewed (TrEMBL) sections of UniProt, based on genera identified from published literature and 16S rRNA gene sequencing-based identification of our samples. This targeted and sample-representative strategy reduced database redundancy, improved peptide to taxon assignment accuracy, and increased protein identification sensitivity. This method provided a more comprehensive reference, encompassing organism-specific proteins, which can be used to improve the accuracy of protein identification. Furthermore, it facilitated the identification of novel or poorly characterised proteins that are often missing in traditional protein research on faecal or gut microbiome [21, 33]. However, this approach has limitations. It's nearly impossible to integrate all information of the microbes present in the gut, as many microbial proteins have not been characterized yet, and most genomic data derived proteins from non-host organisms are unreviewed and have yet to be confirmed in actual biological samples. While this strategy significantly broadens the proteomic landscape, it highlights the need for additional studies to validate these findings within the biological system under investigation.

Our results indicated that fEVs consisted of proteins originating from Kingdoms Bacteria, Animalia (host), Archaea, and Protista, with most from Kingdom Bacteria followed by host. FC samples contained a higher percentage of proteins from Kingdom Bacteria and Kingdom Archaea compared to proteins from the host. However, it is not clearly visible that there is a significant difference between the study groups at the kingdom level.

The comparison between FC and fEVs, highlights the significant difference in fEV proteome compared to FC. Interestingly, host-derived proteins were five times more abundant in fEVs than in FC. This demonstrated the greater utility of fEVs over faeces in studying host-related protein expression in the gut. Particularly, EV-associated proteins offer valuable insights into understanding the communication between host and gut microbiome, making fEVs a more effective tool for these kinds of studies. Most of the enriched host proteins in fEVs were identified as key contributors to maintaining gut defence mechanisms. For instance, proteins such as dipeptidase (DPEP1), cadherin-related family member 5 (CDHR5), olfactomedin 4 (OLFM4), and polymeric immunoglobulin receptor (pIgR) were enriched in fEVs, highlighting the role of fEVs in maintaining gut homeostasis and defence against pathogens [34–39] (Table 1). Proteins involved in secretion and epithelial barrier maintenance, such as Fc gamma binding protein (FCGBP) [40], mucin 13 (MUC13) [41], and calcium-activated chloride channel regulator 1 (CLCA1) [42], were enriched, indicating enhanced gut defence through strengthened mucosal barrier function (Table 1). The enrichment of these epithelial barrier-associated proteins in fEVs can be easily investigated as putative biomarkers for the earliest host response, as the intestinal mucosal barrier serves as the first line of defence against infection.

**Table 1:** Key host proteins significantly upregulated in fEVs compared to FC and their functional roles in gut health

Protein name	Function	Key findings	References
Dipeptidase (DPEP1)	Acts as an adhesion receptor for neutrophil recruitment from the bloodstream into sites of inflammation or injury. Neutrophils serve as the primary defence against invading pathogens and it is essential to recruit neutrophils to the infection site to control the infection effectively	Upregulated in fEVs compared to FC; Reduced in fEVs under infections	[32]
Cadherin related family member 5 (CDHR5)	Facilitates homophilic cell adhesion by binding cadherin molecules on adjacent cells. It plays a critical role in host defence by assembling brush border of intestinal epithelium	Upregulated in fEVs compared to FC; Reduced in fEVs under infections	[33, 34]
Fc gamma binding protein (FCGBP)	improves epithelial defence by promoting the production of mucus in response to pathogenic bacteria, parasites, or viruses that interact with the gut epithelium. This mucus prevents the attachment of pathogens to the mucosal surface and influences pathogen motility	Upregulated in fEVs compared to FC; Reduced in fEVs under infections	[35]
Olfactomedin 4 (OLFM4)	A protein found in gut epithelial cells that controls inflammation and maintains intestinal homeostasis by modulating the immune responses in gut epithelial cells	Upregulated in fEVs compared to FC; Reduced in fEVs under infections	[36]
Polymeric immunoglobulin receptor (pIgR)	The pIgR plays a key role in producing secretory IgA (SIgA), which involves protecting the intestinal epithelium from pathogens. Production of SIgA closely related to the supply of pIgR protein. Furthermore, deficiency of pIgR protein can alter the gut microbiota. Therefore, it is essential in balancing the gut microbiota	Upregulated in fEVs compared to FC	[37, 38]
Mucin 13 (MUC13)	Protecting and maintaining epithelial integrity in the gastrointestinal tract. It contributes to improving the mucosal barrier, modulates immune responses, and supports epithelial cell repair	Upregulated in fEVs compared to FC; Reduced in fEVs under infections	[39]
Calcium-activated chloride channel regulator 1 (CLCA1)	A key regulator of mucus production in the gut and a significant modulator of inflammatory responses, particularly against parasitic nematode infections	Upregulated in fEVs compared to FC; Reduced in fEVs under infections	[40]
Tetraspanins	Mediate the uptake of EVs by recipient cells, influencing intercellular communication, immune responses, and cell signalling	Upregulated in fEVs compared to FC	[41]

Proteins derived from important microbes in maintaining gut defence, such as proteins from *Lactobacillus* spp. were significantly enriched in EVs [43, 44], consistent with previous reports showing that *Lactobacillus*-derived EVs inhibit pathogen colonisation [45]. However, their protein count was reduced in fEVs enriched from *Cryptosporidium*-infected calves, suggesting pathogen-mediated disruption of protective microbial activity may be due to taxonomic dysbiosis. Proteins from *Fibrobacter, Alistipes,* and *Escherichia* were enriched in fEVs. These microbes are known as major GI microbes, contribute to vital gut functions such as plant fiber digestion, nutrient absorption, and energy metabolism. The enrichment of proteins derived from these microbes in fEVs supports fEVs active involvement in these biological processes [44, 46, 47]. For example, previous studies have confirmed that outer membrane vesicles (OMVs) released by *Fibrobacter succinogenes* are enriched with proteins such as carbohydrate-active enzymes. These enzymes are capable of degrading hemicelluloses and pectin, even though *F. succinogenes* itself is unable to utilize non-cellulosic (pentose) sugars for growth [48, 49]. Additionally, *Alistipes,* and *Escherichia* contribute to the production of short-chain fatty acids, which are crucial for the host's energy metabolism [47]. Enrichment of microbial proteins involved in digestion and metabolism in fEVs highlights their potential role in nutrient processing, with evidence of functional contributions from key gut microbes.

Oliveira et al. reported that the bacteria *Fibrobacter, Treponema*, and *Methanobrevibacte* present in the rumen of cattle were not detected in the faeces of the same animals [50]. However, our results demonstrated that even though these bacteria or their markers cannot be detected in faeces, the proteins and EVs they produce can be detected. Thus, fEVs enable the detection of proteins from bacteria while those bacteria are not found in faeces, supporting their value in using fEVs to study microbes present in different sections of the GI tract.

The comparison of fEV protein profiles further revealed substantial reductions in total proteins (53.1%), including host and microbial proteins, in the infected group compared to the healthy group. This highlights the disruptive impact of *Cryptosporidium* infection on the gut microbiota and host metabolism [51], which may lead to the disruption of EV-associated protein secretion and EV-mediated host-microbiota communication. Furthermore, proteins important for maintaining gut immunity and barrier functions, such as DPEP1, CDHR5, OLFM4, FCGBP, MUC13, and CLCA1 proteins, were significantly reduced in the infected group. This suppression could serve as a strategy for pathogens to evade host defence and facilitate colonisation. Furthermore, delivering immunosuppressive molecules via EVs to impair immune responses and hinder clearance as previously reported [13]. These findings demonstrate that the metaproteomic analysis approach we pursued with fEVs not only provides taxonomic information on microbial dysbiosis but also reveals functional dysbiosis in microbial activity, which is more directly relevant to understanding gut physiology and pathophysiology. Although *Cryptosporidium* spp. infection was confirmed in fEV-I group, no parasite-derived proteins were detected in fEV-I group proteomic analysis. This might be due to the very low abundance of *Cryptosporidium* spp. proteins compared with host and microbial proteins, masking them in LC–MS/MS spectra, or the absence of *Cryptosporidium* spp. proteins associated with fEVs due to life cycle status. Particularly, except the oocyst and sporozoite stages, *Cryptosporidium* primarily resides as an intracellular parasite, releasing most of its excretory and secretory products within host cells rather than into the gut lumen. Consequently, parasite-derived EVs or EV-associated proteins are limited to being released into faecal material, and limited extracellular secretions may be rapidly taken up by host and microbial cells. This biological localization could therefore also explain the absence of detectable parasite proteins in the faecal EV proteome. Moreover, none of the animals exhibited obvious clinical symptoms of cryptosporidiosis, suggesting that infections were mostly at a subclinical stage. At this stage, parasite load and secreted protein levels are typically low, which may further explain the absence of detectable parasite proteins in the fEV proteome. Therefore, further investigations are necessary to elucidate this phenomenon and to refine methodologies for the detection of parasite-derived proteins associated with fEVs.

We propose that analysing fEVs offers a powerful, non-invasive approach for comprehensive profiling of the GI microbiota. Traditional faecal microbiota analyses primarily reflect microbial populations from the distal GI tract, limiting their ability to capture the full diversity of gut communities [50]. In contrast, fEVs contain proteins from microbes inhabiting all regions of the GI tract, providing functional insights into hard-to-access areas (Fig. S1). For instance, proteins from forestomach-residing microbes such as *Fibrobacter, Treponema*, and *Methanobrevibacter*, rarely detected in faeces [50] were identified in fEVs [1, 52, 53], highlighting their broader spatial representation. The fEVs also carried proteins from microbes involved in key gut functions, such as *Prevotella, Fibrobacter, Treponema*, and *Actinomyces* (plant fiber digestion) or early rumen colonisers like *Enterococcus, Streptococcus, Parabacteroides*, and *Lactococcus* [30, 54], and small intestine-associated taxa such as *Clostridium, Candidatus, Bifidobacterium, Lactobacillus*, and *Eubacterium* (protein metabolism and beneficial probiotic-associated functions). Additionally, proteins from large intestine-associated microbes like *Alistipes* and *Bifidobacterium* are important for mucus layer maintenance, and proteins from these microbes were also detected in fEVs [1, 43, 55–57]. These findings underscore the utility of fEVs in capturing microbial activities in different regions of the GI tract and their potential to advance microbiome research beyond the limitations of conventional methods.

Even with the limited sample numbers analysed plus limited curated annotations for bovine gut fEV proteome, our approach revealed distinct potential functional annotation profiles between healthy and *Cryptosporidium* spp. infected calves, highlighting either the selective packaging of proteins into EVs to support specific microbial and host functions, or alternatively, the presence of different microbial populations secreting EVs with particular protein cargo adapted for distinct functional roles. In healthy animals, fEVs were enriched with host proteins involved in specialised activities such as actin binding, guanyl-nucleotide exchange factor activity, calcium ion binding, and metallopeptidase functions, reflecting roles in cytoskeletal regulation, signalling, ion transport, and enzymatic remodelling. Similarly, fEVs were enriched with microbial proteins associated with ATP binding, DNA and nucleic acid interactions, and metal ion binding (notably zinc), reflecting roles in energy metabolism, gene regulation, and enzymatic processes. Hydrolase and transferase activities further pointed to involvement in macromolecule breakdown, signalling, and intracellular communication. In contrast, infection led to a marked depletion of microbial proteins in fEVs related to ATPase activities, DNA binding, and transcriptional regulation, indicating a reduction in fEV-mediated functions linked to energy metabolism and gene regulation. Endonuclease, kinase, and nucleic acid binding activities that were present under healthy conditions were completely absent in infection, further supporting impaired nucleic acid processing and signalling capacity. However, transferase activity and stress-response functions (e.g., oxidoreductase, magnesium ion binding) were enriched in the infected group, indicating an adaptive microbial response to infection-driven oxidative stress and metabolic disruption.

Annotated proteins under potential biological processes showed that fEVs are enriched with host proteins involved in structural processes, such as intermediate filament organisation and keratinisation. They are also enriched in regulatory and signalling processes, including Rho protein signal transduction, calcium ion transport, cell adhesion, lipid metabolism, proteolysis, and ruffle assembly, suggesting roles in dynamic cell communication and barrier function. Similarly, fEVs are carrying microbial proteins specialised for important biological processes such as DNA maintenance, cell wall organisation, protein processing, and energy and nutrient metabolism, indicating selective packaging of proteins with regulatory, signalling, and adaptive functions. Notably, proteins related to peptidoglycan biosynthesis were elevated in infected animals, which may reflect microbial adaptive responses to the altered gut environment and heightened host immune activity during *Cryptosporidium* spp. infection. Together, all these analyses demonstrate that fEVs proteomics can be studied to understand dynamic responses to infections in the gut by modulating microbial and host functions, supporting microbial survival and potentially influencing gut ecosystem stability.

To understand the impact of lyophilization without protective agents on protein composition and relative abundance in fEVs, we compared proteomic profiles of lyophilised and frozen samples. Lyophilisation without any cytoprotectants introduced measurable changes to the fEV proteome. In particular, we observed a marked loss of host-derived proteins, indicating that these proteins are unstable under lyophilised conditions. This instability suggests that lyophilised fEVs without lyoprotectants may be unsuitable for investigating host-related physiology or pathophysiology [58]. Thus, our results suggest the need for further studies using lyoprotectants like DMSO, trehalose, sucrose, and mannitol addition that may preserve sufficient proteomic integrity to be considered as a viable storage method for fEVs, which has been successfully done with other therapeutic EVs types [58].

Although the sample size in this study was limited, the observed significant differences have a large effect size, which provides strong preliminary evidence for subsequent larger-scale studies. Moreover, many of the microbial proteins identified using our customised database remain unreviewed, however, use of unreviewed UniProt data expanded the detectable protein landscape but introduced challenges in annotation confidence. Therefore, these proteins require further biological validation for exact functional analysis, yet we are demonstrating the possibility of employing metaproteomic with fEVs as a novel tool with larger-scale validation.

In future studies, we can expand the sample sources to include calves of different ages, breeds, and management systems across diverse geographic regions to verify whether the identified core fEV protein biomarkers are consistent and universal indicators of *Cryptosporidium* infection. Validation using independent cohorts and targeted assays such as ELISA or targeted MS will be essential to confirm their diagnostic reliability and promote translation into practical diagnostic reagents suitable for on-farm or clinical applications. In addition, integrating fEV proteomics with transcriptomic and metabolomic analyses to construct a comprehensive multi-omics network will help uncover the coordinated molecular mechanisms through which fEVs regulate host-microbiome-pathogen interactions during intestinal infections. Furthermore, exploring the protective effects of lyoprotectants on the structural and proteomic integrity of fEVs during lyophilisation and storage will be valuable for developing standardised protocols that ensure the stability and long-term preservation of fEVs for diagnostic and functional applications. Together, these directions will advance the translation of fEV-based discoveries into robust, clinically applicable tools for monitoring gut health and infectious diseases in livestock.

## Conclusion

In conclusion, we demonstrated that fEVs associated with host and microbial proteins can serve as a valuable tool to explore gut physiology, pathophysiology, host-microbial-pathogen interactions, and gut microbiome changes. The shifts of proteome profiles emphasise the ability of the pathogen to manipulate host immune defences and microbiome composition for its survival and replication. Further, fEVs proteins also reflect the potential of describing both taxonomic and functional dysbiosis from the same sample type. Overall, this study establishes foundational evidence for the greater potential of fEVs as a powerful tool in biomarker discovery and for investigating gastrointestinal health and diseases.

## Supplementary Information

Additional file 1: Table S1. List of microbes identified in calf faeces based on 16S rRNA gene amplicon sequencing.

Additional file 2: Fig. S1. Microbial genera identified based on fEV proteome and the regions they are highly abundance in the gastro intestinal tract.

## Data Availability

Mass spectrometry data are available in ProteomeXchange Consortium via the PRIDE with the dataset identifier PXD068377. The additional data that support the findings of this study are available from the first and corresponding author, upon reasonable request.

## Abbreviations

BCV: Bovine coronavirus
BP: Biological processes
CC: Cellular component
EVs: Extracellular vesicles
FC: Faecal crude
fEV-H: Faecal extracellular vesicles from healthy calves
fEV-I: Faecal extracellular vesicles from *Cryptosporidium *spp. infected calves
fEV-L: Lyophilized faecal extracellular vesicles
fEV-NL: Non-lyophilized faecal extracellular vesicles
fEVs: Faecal extracellular vesicles
GI: Gastrointestinal
GO: Gene Ontology
LC–MS/MS: Liquid chromatography/mass spectrometry-mass spectrometry
MF: Molecular function
NTA: Nanoparticle tracking analysis
PBS: Dulbecco’s phosphate-buffered saline
PCA: Principal component analysis
SEC: Size exclusion chromatograph
TEM: Transmission electron microscopy
VSN: Variance stabilizing normalization
WB: Western blotting
