# Supplementary material for: Cryptosporidium spp. infection drives distinct alterations in the faecal extracellular vesicles metaproteome of calves
Source: J Anim Sci Biotechnol. 2026 Jan 15;17:8. doi: 10.1186/s40104-025-01332-4 (PMC12805734; doi:10.1186/s40104-025-01332-4)
Supplement: Supplementary file 2 — Additional file 2: Fig. S1. Microbial genera identified based on fEV proteome and the regions they are highly abundance in the gastro intestinal tract. [file 40104_2025_1332_MOESM2_ESM.docx]

| **GIT Region** | **Microbial genera associated with detected proteins** | **Notes** | **References** |
| --- | --- | --- | --- |
| **Stomach (Rumen, Reticulum, Omasum, Abomasum)** | *Bifidobacterium, Eubacterium, Fibrobacter, Parabacteroides, Paraprevotella, Prevotella, Ruminococcus, Treponema, Bifidobacterium, Treponema, Methanobrevibacter, Butyrivibrio, Candidatus, Methanosphaera, Actinomyces, Enterococcus, Streptococcus, Lactococcus* | Genera involved in fiber digestion and fermentation of complex carbohydrates. | ^1–6^ |
| **Small Intestine** | *Bifidobacterium, Clostridium, Eubacterium, Lactobacillus, Prevotella, Streptococcus, Escherichia, Lachnoclostridium, Methanobrevibacter, Methanosphaera, Methanocorpusculum, Romboutsia, Staphylococcus, Candidatus* | Bacteria adapted to nutrient-rich environments and moderate oxygen levels. Important for cellulose degradation and utilization, breakdown the proteins from diet, producing probiotics | ^1,3,5–7^ |
| **Large Intestine** | *Alistipes, Blautia, Clostridium, Coprococcus, Eubacterium, Faecalibacterium, Lactobacillus, Parabacteroides, Oscillibacter, Flavonifractor, Lachnoclostridium, Methanobrevibacter, Methanocorpusculum, Methanosphaera, Parabacteroides, Roseburia, Treponema, Bifidobacterium* | Genera specialize in breaking down remaining fibers and producing short-chain fatty acids | ^1,5,6^ |


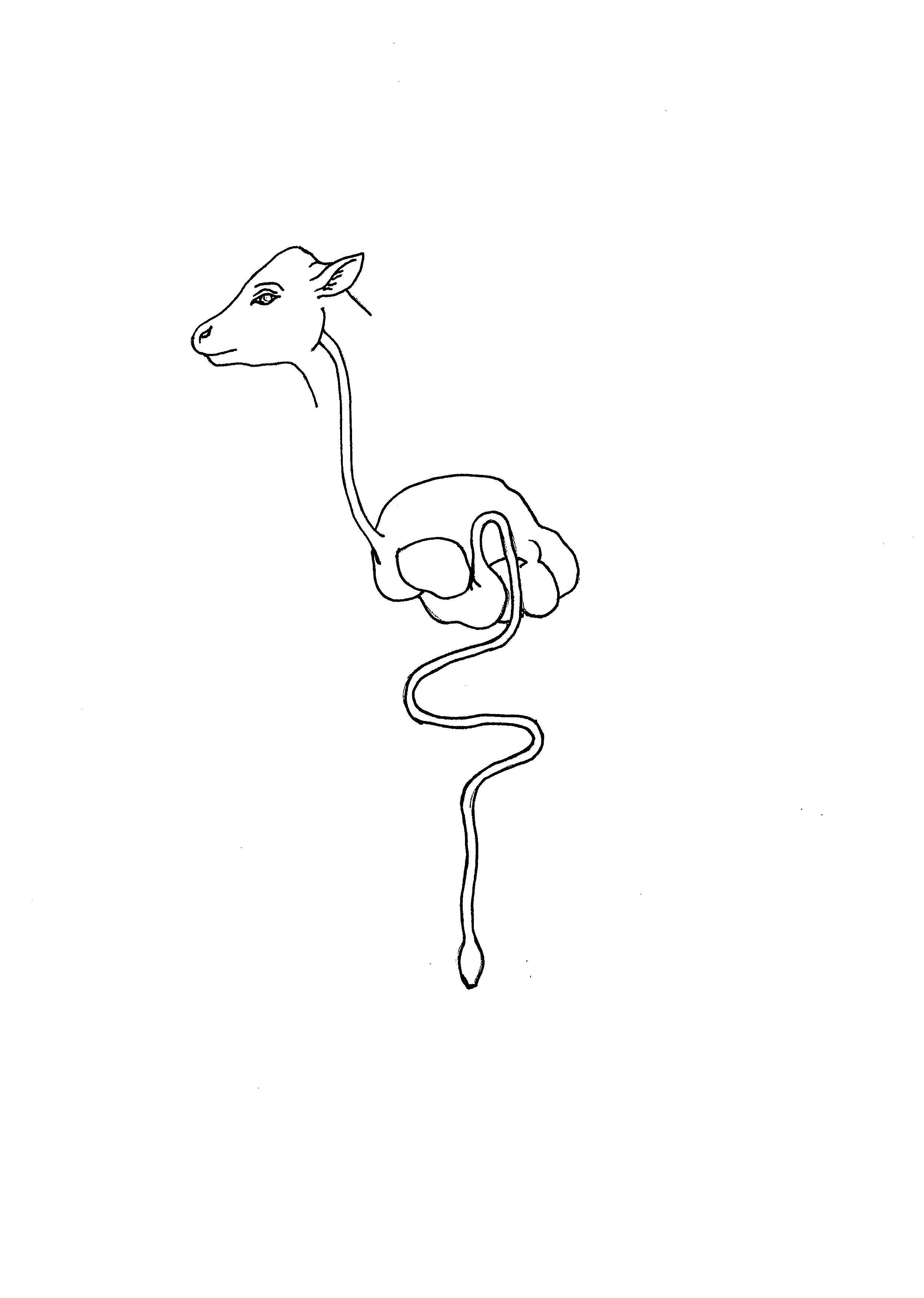


Figure:

**Supplementary Figure 1:** Microbial genera identified based on fEV proteome and the regions they are highly abundance in the gastro intestinal tract.

References

1. Du Y, Gao Y, Hu M, et al. Colonization and development of the gut microbiome in calves. *J Anim Sci Biotechnol*. 2023;14(1). doi:10.1186/s40104-023-00856-x

2. Hartinger T, Pacífico C, Poier G, Terler G, Klevenhusen F, Zebeli Q. Shift of dietary carbohydrate source from milk to various solid feeds reshapes the rumen and fecal microbiome in calves. *Sci Rep*. 2022;12(1). doi:10.1038/s41598-022-16052-2

3. Xu Q, Qiao Q, Gao Y, et al. Gut Microbiota and Their Role in Health and Metabolic Disease of Dairy Cow. *Front Nutr*. 2021;8. doi:10.3389/fnut.2021.701511

4. Li K, Shi B, Na R. The Colonization of Rumen Microbiota and Intervention in Pre-Weaned Ruminants. *Animals*. 2023;13(6). doi:10.3390/ani13060994

5. Dias J, Inácio Marcondes M, Motta de Souza S, et al. *Bacterial Community Dynamics across the Gastrointestinal Tracts of Dairy Calves during Preweaning Development*.; 2018. https://journals.asm.org/journal/aem

6. Lin L, Lai Z, Zhang J, Zhu W, Mao S. The gastrointestinal microbiome in dairy cattle is constrained by the deterministic driver of the region and the modified effect of diet. *Microbiome*. 2023;11(1). doi:10.1186/s40168-022-01453-2

7. Malmuthuge N, Liang G, Griebel PJ, Guan LL. Taxonomic and functional compositions of the small intestinal microbiome in neonatal calves provide a framework for understanding early life gut health. *Appl Environ Microbiol*. 2019;85(6). doi:10.1128/AEM.02534-18
